# Personality disorder symptomatology is associated with anomalies in striatal and prefrontal morphology

**DOI:** 10.3389/fnhum.2015.00472

**Published:** 2015-08-31

**Authors:** Doris E. Payer, Min Tae M. Park, Stephen J. Kish, Nathan J. Kolla, Jason P. Lerch, Isabelle Boileau, M. M. Chakravarty

**Affiliations:** ^1^Addictions Program, Centre for Addiction and Mental Health, TorontoON, Canada; ^2^Research Imaging Centre, Centre for Addiction and Mental Health, TorontoON, Canada; ^3^Department of Psychiatry, University of Toronto, TorontoON, Canada; ^4^Cerebral Imaging Centre, Douglas Mental Health University Institute, VerdunQC, Canada; ^5^Schulich School of Medicine and Dentistry, Western University, LondonON, Canada; ^6^Complex Mental Illness Program, Forensic Service, Centre for Addiction and Mental Health, TorontoON, Canada; ^7^Department of Medical Biophysics, University of Toronto, TorontoON, Canada; ^8^Mouse Imaging Centre, Hospital for Sick Children, TorontoON, Canada; ^9^Department of Psychiatry and Biomedical Engineering, McGill University, MontrealQC, Canada

**Keywords:** personality, DSM Axis-II, magnetic resonance imaging, morphology, striatum

## Abstract

Personality disorder symptomatology (PD-Sx) can result in personal distress and impaired interpersonal functioning, even in the absence of a clinical diagnosis, and is frequently comorbid with psychiatric disorders such as substance use, mood, and anxiety disorders; however, they often remain untreated, and are not taken into account in clinical studies. To investigate brain morphological correlates of PD-Sx, we measured subcortical volume and shape, and cortical thickness/surface area, based on structural magnetic resonance images. We investigated 37 subjects who reported PD-Sx exceeding DSM-IV Axis-II screening thresholds, and 35 age, sex, and smoking status-matched control subjects. Subjects reporting PD-Sx were then grouped into symptom-based clusters: *N* = 20 into Cluster B (reporting Antisocial, Borderline, Histrionic, or Narcissistic PD-Sx) and *N* = 28 into Cluster C (reporting Obsessive–Compulsive, Avoidant, or Dependent PD-Sx); *N* = 11 subjects reported PD-Sx from both clusters, and none reported Cluster A (Paranoid, Schizoid, or Schizotypal) PD-Sx. Compared to control, Cluster C PD-Sx was associated with greater striatal surface area localized to the caudate tail, smaller ventral striatum volumes, and greater cortical thickness in right prefrontal cortex. Both Cluster B and C PD-Sx groups also showed trends toward greater posterior caudate volumes and orbitofrontal surface area anomalies, but these findings did not survive correction for multiple comparisons. The results point to morphological abnormalities that could contribute to Cluster C PD-Sx. In addition, the observations parallel those in substance use disorders, pointing to the importance of considering PD-Sx when interpreting findings in often-comorbid psychiatric disorders.

## Introduction

Personality disorders [PD; Axis II of the DSM-IV (APA, 2000)] are enduring, pervasive, and inflexible patterns of inner experience and behavior that deviate from cultural expectations and lead to distress or impairment. Even in the absence of a formal PD diagnosis, personality traits reflecting PD symptomatology (PD-Sx) can lead to emotional and interpersonal problems (e.g., Daley et al., 2000; Rosenthal et al., 2005; Dick et al., 2013; Gervais et al., 2013), and are often comorbid with major psychiatric conditions (DSM-IV Axis I disorders), particularly substance use, mood, and anxiety disorders (Daley et al., 1999; Chabrol et al., 2004; Hallquist and Lenzenweger, 2013). For this reason, PD symptoms are more prevalent in psychiatric populations (Bagby et al., 2008; Grant et al., 2012), and can present risk factors for emergence of additional psychiatric disorders and/or barriers to treatment. A better understanding of PD-Sx can therefore improve not only the management of PDs, but potentially also that of comorbid psychiatric disorders.

Personality disorders pathology has traditionally been grouped into three symptom-based clusters (an approach that has clinical utility and was recently confirmed empirically, Cox et al., 2012): Cluster A (odd/eccentric), including Paranoid, Schizoid, and Schizotypal PD; Cluster B (dramatic/emotional/erratic), including Antisocial, Borderline, Histrionic, and Narcissistic PD; and Cluster C (anxious/fearful), including Obsessive–Compulsive, Avoidant, and Dependent PD. Despite the shared symptomatology within clusters, however, shared neurobiology has not been extensively investigated.

Cluster B personality traits are characterized by emotion dysregulation, poor response inhibition, and impulsive and externalizing behaviors, and are strongly associated with substance use disorders (Charney et al., 2010; Jahng et al., 2011; Ersche et al., 2012). Accordingly, neuroimaging studies examining Cluster B PD-Sx (Matsuo et al., 2009; Bjork et al., 2010; Schilling et al., 2013; Castellanos-Ryan et al., 2014), as well as diagnosed Cluster B PDs (Borderline and Antisocial PD; BPD, ASPD), have found abnormalities in fronto-limbic circuitry, including ventral striatum, amygdala, and hippocampus, along with orbitofrontal and prefrontal cortex (OFC, PFC), cingulate cortex, and insula (Anderson and Kiehl, 2012; O’Neill and Frodl, 2012; Sato et al., 2012; Frodl and Skokauskas, 2014). Of note, whereas cortical and limbic regions are often associated with *deficits* in structure and function (Matsuo et al., 2009; Nunes et al., 2009; Yang and Raine, 2009; Ruocco et al., 2012; Schulze et al., 2013; Castellanos-Ryan et al., 2014), striatal volumes tend to be *enlarged* in individuals with Cluster B PD-Sx (Ersche et al., 2012) and Cluster B PDs (Brambilla et al., 2004; Glenn and Yang, 2012^1^). This parallels, and could potentially contribute to, the frequent finding of striatal enlargement in stimulant addiction (Chang et al., 2007; Kish, 2011; Mackey and Paulus, 2013).

Cluster C personality traits are characterized by a focus on avoiding the experience of anxiety, and are also highly comorbid with mood, anxiety, and substance use disorders (DeJong et al., 1993; Preuss et al., 2009; Langås et al., 2012). A Cluster C PD, obsessive–compulsive PD (OCPD), is the most prevalent PD in the US population (∼8%, Grant et al., 2012), yet little is known about the neurobiology underlying its traits. However, like Axis I Obsessive–Compulsive Disorder (OCD), rigid behavioral patterns implicate cortico-basal-thalamic loops involved in cognitive flexibility and behavioral adaptation (Fineberg et al., 2010). In line with this view, recent studies of individuals with compulsive traits showed enlarged striatal and OFC/PFC volumes (Ersche et al., 2012; Montigny et al., 2013), paralleling findings from OCD patients and their unaffected siblings (Shaw et al., 2015). Beyond compulsivity, however, the neurobiology of Cluster C traits remains largely unexplored.

The present study used magnetic resonance imaging (MRI) in combination with a novel automated method for subcortical segmentation and surface-based shape analysis (MAGeT Brain, Chakravarty et al., 2013, 2015; Raznahan et al., 2014) to examine brain morphological features associated with Cluster B and C PD-Sx. Based on the fronto-striatal abnormalities recently revealed using the same methodology in individuals with OCD and their unaffected siblings (Shaw et al., 2015), and the above-reviewed findings of cortical thinning and striatal enlargement in Cluster B PDs, we examined the striatum and PFC/OFC of PD-symptomatic individuals, with the hypothesis that cluster-level differences echoing findings in PDs would emerge.

## Materials and Methods

### Participants

All procedures were approved by the Centre for Addiction and Mental Health Research Ethics Board (Toronto, ON, Canada), and complied with ethical standards of the Declaration of Helsinki. Volunteers were recruited from the community through flyers and Internet advertisements. After complete description of the study, all volunteers gave written informed consent.

Volunteers completed screening for study inclusion/exclusion with the following criteria: (1) 18–55 years old; (2) no current Axis I disorder (as per SCID for DSM-IV, First et al., 2002); (3) no lifetime history of alcohol or substance dependence (except nicotine) and no recent use of recreational drugs, confirmed with urine and hair analysis (light cannabis use was permitted); (4) no medical conditions likely to affect the brain; (5) no current use of psychotropic medications; and (6) no MR contraindications. The sample described here has previously been included in other published articles (Kish et al., 2010; Boileau et al., 2012, 2013; Payer et al., 2013), where they formed the comparison groups in studies of addiction.

### Measures and Procedures

For each subject, PD-Sx was assessed using the SCID-II Personality Questionnaire (SCID-II/PQ, First et al., 1997), which has confirmed validity and reliability, and is widely used in personality research (Ball et al., 2001). The questionnaire consists of 119 yes/no questions describing diagnostic features of each PD (as per the DSM). Endorsing a criterion number of items (i.e., exceeding a symptom threshold defined by the DSM) results in a positive screen for a given PD. Although a positive screen indicates a possible PD diagnosis, a follow-up clinical interview is necessary to finalize the diagnosis; the questionnaire alone only establishes (potentially clinically significant) symptomatology (i.e., PD-Sx).

Based on SCID-II Personality Questionnaire results, study participants were divided into those endorsing a number of symptoms exceeding threshold for at least one PD (PD-Sx group) and a Control group endorsing no symptoms exceeding threshold for any PD. The PD-Sx group was then further divided into those endorsing Cluster B and/or Cluster C PD-Sx (**Table 1**).

**Table 1:** Participant characteristics.

	HC (*N* = 35)	PD (*N* = 37)	Comparison PD v HC	Cluster B (*N* = 20)	Comparison B v HC	Cluster C (*N* = 28)	Comparison C v HC	BC-Comorbid (*N* = 11)	Comparison BC v HC
Age (years)	30.9 ± 9.4	27.5 ± 9.4	*p* = 0.12	27.8 ± 9.3	*p* = 0.23	27.5 ± 9.0	*p* = 0.14	27.9 ± 8.4	*p* = 0.34
Sex	16F/19M	11F/26M	*p* = 0.22	8F/12M	*p* = 0.78	7F/21M	*p* = 0.12	4F/7M	*p* = 0.73
Education (years)	16.5 ± 2.1	15.5 ± 1.9	***p* = 0.02**	15.5 ± 1.8	***p* = 0.053**	15.6 ± 2.0	***p* = 0.07**	15.8 ± 1.9	*p* = 0.31
Current smokers (N)	5	11	*p* = 0.16	5	*p* = 0.47	9	*p* = 0.13	3	*p* = 0.37
Cannabis smokers (N) (past 30 days)	4	4	*p* = 1.00	4	*p* = 0.44	3	*p* = 1.00	3	*p* = 0.33
Total Brain Volume^a^	1.04e6 ± 0.12e6	1.12e6 ± 0.11e6	*p* = 0.05	1.10e6 ± 0.13e6	*p* = 0.15	1.13e6 ± 0.13e6	***p* = 0.04**	1.13e6 ± 0.13e6	*p* = 0.11
PD Symptoms Endorsed (N)	–	–	–	Antisocial (2), Narcissistic (15), Borderline (4), Histrionic (6)		Avoidant (4), Obsessive-Compulsive (25)		Obsessive-Compulsive (all) + Narcissistic (5); + Antisocial (1); + Histrionic (1); + Narcissistic + Borderline (2); + Avoidant + Narcissistic (1); + Histrionic + Narcissistic (1)	

*^a^Analysis adjusted for age and sex; HC, Healthy Control group; PD, Personality Disorder symptomatic group. Bold values represent significant or approaching significance at α = 0.05.*

Brain images were acquired on a Signa 1.5 Tesla MRI scanner (General Electric Medical Systems, Milwaukee, WI, USA), using a high-resolution T1-weighted spoiled gradient recalled acquisition sequence. Scan parameters were: repetition time = 8.9–12 ms; echo time = 5.3–15 ms, flip angle = 45°; slice thickness = 1.5 mm, 0 gap; 124 slices; field of view 22 cm × 16 cm; matrix = 256 × 256, resulting in 1.5 mm × 0.78 mm × 0.78 mm voxels.

### Image Processing

#### Subcortical Segmentation

Striatal morphology was estimated using MAGeT Brain (Chakravarty et al., 2013, 2015; Raznahan et al., 2014), a novel multi-atlas technique that bootstraps segmentation using Multiple Automatically Generated Templates. This technique has been optimized and validated for striatal structures, so that analyses focused on bilateral caudate, putamen, and ventral striatum (nucleus accumbens). However, since a recent variant of the MAGeT Brain algorithm was shown to reliably segment the hippocampus and amygdala using additional high-resolution atlases as input (Winterburn et al., 2013; Pipitone et al., 2014; Treadway et al., 2014), these structures were additionally examined in exploratory analyses.

All segmentations were manually checked by an expert observer (MTMP) prior to analysis. Outcome measures were (voxel-based) *volume* and vertex-wise measures of *shape* and *surface area* (SA; only volume was available for the exploratory hippocampus and amygdala analyses). Shape is measured as a series of surface displacement metrics that describe the inward or outward displacement along a surface normal required for the atlas (Chakravarty et al., 2006) to match each subject (see Chakravarty et al., 2015; Janes et al., 2015) and corresponding local SA differences (as recently reported in Raznahan et al., 2014 and Shaw et al., 2015). SA at each vertex was divided by the total SA of each given structure in an individual to account for global effects of volume on local vertex-wise measures.

For volume normalization, total brain volume (TBV) was obtained using the brain extraction based on non-local segmentation technique (BEaST) pipeline (Eskildsen et al., 2012), which allows for accurate and robust brain extraction.

#### Cortical Measures

To determine prefrontal/orbitofrontal contributions to PD-Sx, *cortical thickness* (CT) and *cortical SA* were estimated on the T1-weighted images using the fully automated CIVET 1.1.10 pipeline (Lyttelton et al., 2007). In brief, the images were linearly registered to standard stereotaxic space defined by the MNI ICBM 152 model (Collins et al., 1994; Mazziotta et al., 2001). The images were then corrected for intensity non-uniformity using non-parametric non-uniform intensity normalization (N3, Sled et al., 1998) and a non-linear registration to the model was applied. Tissue classification was then performed using INSECT (Zijdenbos et al., 1998), classifying each voxel as white matter (WM), gray matter (GM), or cerebrospinal fluid (CSF). The images were then mapped to a probabilistic atlas using the ANIMAL algorithm (Automatic Non-linear Image Matching and Anatomical Labeling, Collins et al., 1995). Finally, the WM surface was generated by using an ellipsoid polygonal model that deforms to fit the WM/GM interface and the pial surface (Lerch and Evans, 2005). To generate the GM surface, the WM surface was expanded until it reached the GM/CSF interface (Kim et al., 2005). The resulting surfaces were composed of 40,962 vertices for each hemisphere, and CT was estimated as the distance, in mm, between homologous vertices in the WM and GM matter surfaces. SA was estimated at each vertex as the average value of all adjoining vertices. CT and SA data were blurred using a surface-based diffusion smoothing kernels of 20 and 40 mm full-width at half-maximum (FWHM), respectively, to preserve the concordance between quantitative values and cortical topology (Chung et al., 2003) and non-linearly aligned with a surface-based registration (Lyttelton et al., 2007).

Region-of-interest (ROI)-based CT and SA were also estimated, using the intersection of the cortical surfaces estimated by CIVET (above) and the LPBA40 atlas (Shattuck et al., 2008), resulting in a total of 40 cortical regions per hemisphere providing a single output value per region (see, e.g., Wheeler et al., 2015). Among these, we focused on *a priori* PFC and OFC ROIs, as these have been consistently linked to PD-Sx, along with additional exploratory analyses in less consistently identified but potentially relevant ROIs: cingulate cortex and insula.

### Statistical Analysis

To examine differences in striatal and cortical morphology, vertex-wise analyses were conducted using the RMINC package (https://github.com/Mouse-Imaging-Centre/RMINC). Prior to analysis of subcortical structures, SA at each vertex was divided by the total SA of the given structure to account for global effects. A general linear model including Cluster B and Cluster C PD-Sx as factors (specifying presence/absence of symptomatology exceeding threshold for a PD in that cluster), and age, sex, education, and smoking status as covariates, was applied at each vertex. Covariates were included because they were previously shown to impact morphometry (particularly smoking, see Janes et al., 2015; Vafaee et al., 2015; also see Raz et al., 1995) and/or differed between groups in our sample (see **Table 1**). Follow-up analyses then individually compared Cluster B and C PD-Sx groups to the Control group. Results were corrected for multiple comparisons using false discovery rate (FDR) correction (Genovese et al., 2002).

To complement the vertex-wise analysis, volume/SA in subcortical structures and CT/SA in cortical ROIs were entered into SPSS, and analyzed using omnibus MANCOVAs paralleling the vertex-wise analysis (i.e., Cluster B and C PD-Sx as factors, and age, sex, education, smoking status, and TBV [for subcortical volume and cortical SA] as covariates). Two follow-up ANCOVAs then individually compared Cluster B and C PD-Sx groups to the Control group. Analyses were corrected for multiple comparisons to account for the number of striatal subregions (bilateral anterior and posterior caudate and putamen, and ventral striatum) and the number of PFC/OFC ROIs (bilateral inferior, middle, and superior frontal gyrus; medial and lateral orbitofrontal cortex), using FDR correction (Benjamini and Hochberg, 1995). Given previous but less consistent associations with PD-Sx, exploratory analyses additionally examined effects in the anterior cingulate cortex and insula.

## Results

### Participant Characteristics

A total of 72 participants were included in the study, see **Table 1** for demographic characteristics. Of the 72 participants, 37 endorsed symptoms exceeding threshold for at least one PD (PD-Sx group): 20 endorsed symptoms consistent with a Cluster B PD, and 28 endorsed symptoms consistent with a Cluster C PD; of these, 11 endorsed symptoms from both Clusters B and C. None of the participants endorsed a criterion number of symptoms for Cluster A. The remaining 35 subjects did not self-report any symptoms meeting PD thresholds, and comprised the control group. The two groups were matched for age, sex, current cigarette smoking status, and past-month cannabis use, but subjects in the PD-Sx group had completed fewer years of education (15.5 vs. 16.5 years, *p* = 0.02) and had greater brain volumes (adjusted for age and sex, *p* = 0.05). Education and TBV were therefore included as covariates in analyses, as described in Section “Statistical Analysis.”

### Striatal Morphology

**FIGURE 1:**
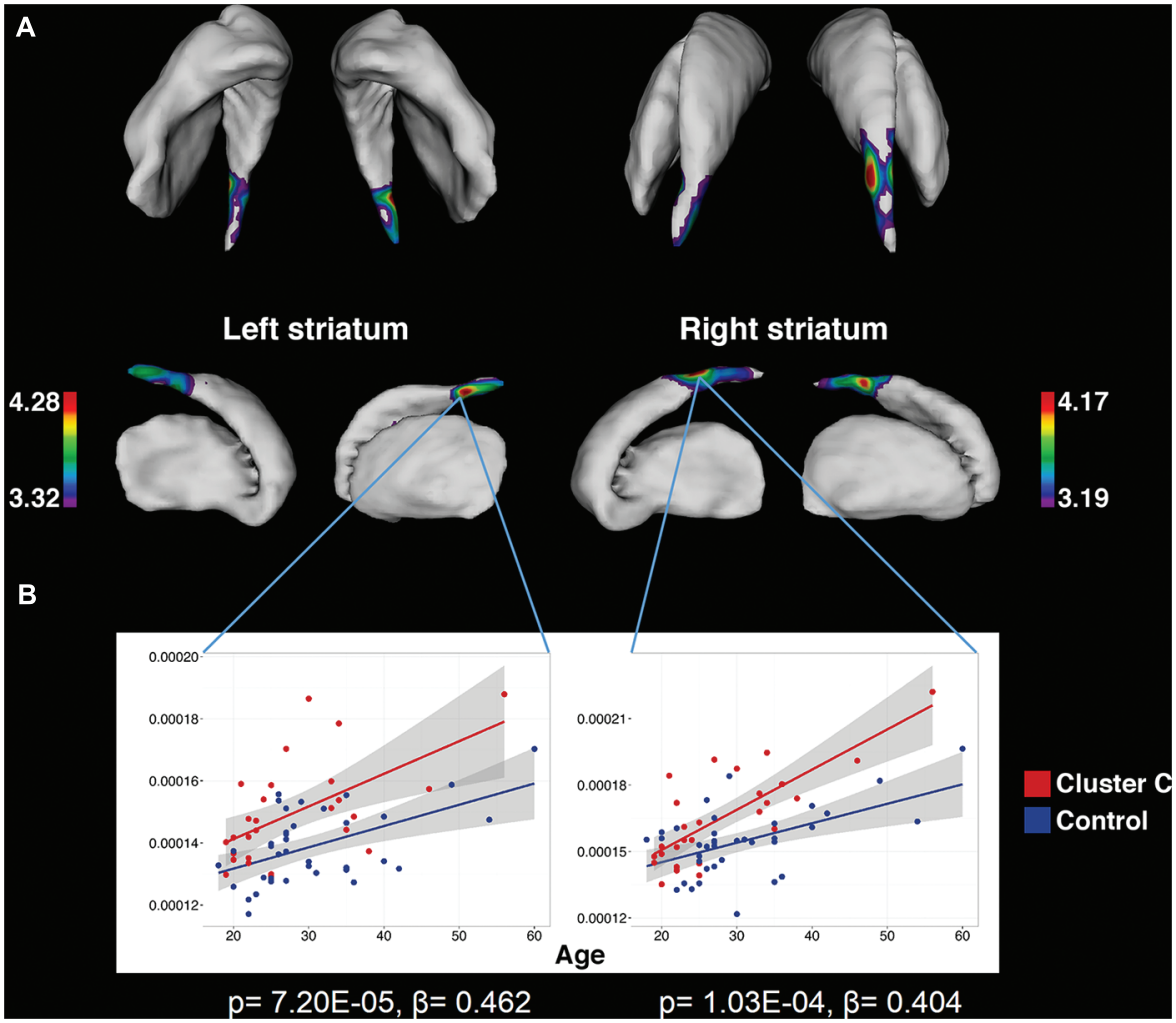
**Significant associations between Cluster C symptomatology and greater striatal surface area, localized to the caudate tail, as identified by vertex-wise analysis. (A)** Colored regions indicate vertices with significant *t*-statistics (as noted by the color bar) after FDR correction (*q* = 0.05). **(B)** To demonstrate that the observed differences are persistent across the age range and do not interact with age, surface area is plotted against age at vertices of peak significance. Statistics indicate results of multiple linear regression at peak vertices, with effects of Cluster C noted by *p* and β.

Vertex-wise analysis in the striatum revealed greater regional SA localized to the caudate tail in individuals with Cluster C PD-Sx when compared to Control (**Figure 1**). In addition, the volumetric analysis revealed significant effects in ventral striatum, reflecting lower volumes in the Cluster C PD-Sx compared to the Control group (right *p* = 0.007, left *p* = 0.043; **Table 2**).

**Table 2:** Striatal volume and cortical thickness effects associated with Cluster C PD-Sx.

	Omnibus analysis^a^		Cluster C vs. Control analysis			
	Cluster C effect		Marginal mean (SE)			
	*p*	Cohen’s *d*	Control (*N* = 35)	Cluster C (*N* = 28)	*p*	$\eta_{p}^{2}$
**Striatal volume**						
Ventral striatum (R)	**0.01^b^**	0.02	892.60 (11.35)	841.60 (12.88)	**0.007^b^**	0.122
Ventral striatum (L)	0.06	0.12	1045.11 (13.74)	999.48 (15.59)	**0.043**	0.071
**Cortical thickness**						
Middle frontal gyrus (R)	**0.01^b^**	0.72	9.52 (0.07)	9.81 (0.08)	**0.016^b^**	0.097
Superior frontal gyrus (R)	**<0.001^b^**	0.80	3.41 (0.03)	3.51 (0.03)	**0.017^b^**	0.096
Lateral OFC (R)	**0.04**	0.51	3.49 (0.04)	3.62 (0.04)	**0.028^b^**	0.082
Inferior frontal gyrus (R)	**0.05**	0.50	3.50 (0.03)	3.56 (0.03)	0.118	–
Superior frontal gyrus (L)	**0.03**	0.56	3.45 (0.03)	3.53 (0.03)	0.054	–

*^a^Model includes Cluster B and Cluster C as factors, and age, sex, education, smoking status, and total brain volume (for striatum analysis) as covariates; ^b^Survives false-discovery-rate correction for multiple comparisons. L, left; R, right; OFC, orbitofrontal cortex. Bold values represent significant or approaching significance at α = 0.05.*

We found no significant effects associated with Cluster B PD-Sx, although a trend emerged for posterior caudate enlargement (*p* = 0.02, uncorrected; see Supplementary Materials).

### Cortical Morphology

In PFC/OFC, the ROI-based omnibus MANCOVA identified CT effects associated with Cluster C PD-Sx in right PFC, reflecting greater CT in the Cluster C PD-Sx group than Control (all *p* < 0.028; **Table 2**). This pattern was reflected in the vertex-wise analysis at a relatively lenient threshold (see Supplementary materials), but did not survive FDR correction at *q* = 0.05.

No CT effects were observed for Cluster B PD-Sx, although the ROI-based omnibus MANCOVA analyzing SA pointed to an effect of Cluster B PD-Sx in lateral orbitofrontal cortex (*p* = 0.02, uncorrected), reflecting lower SA in Cluster B PD-Sx than Control subjects (*p* = 0.036, uncorrected; Cohen’s *d* = 0.62; see Supplementary Table S1); however, these effects did not survive correction for multiple comparisons across *a priori* ROIs.

### Exploratory Analyses

No other results survived correction for multiple comparisons. However, all relevant comparisons and effect size estimates are presented in Supplementary Table S1. Exploratory results include greater posterior caudate volumes in both Cluster B and C PD-Sx groups compared to control, along with greater hippocampal volumes. Amygdala volumes showed no differences from control in either PD-Sx group. For cortical measures, results revealed greater cingulate CT in Cluster C PD-Sx, and lower lateral but greater medial orbitofrontal SA in both Cluster B and C PD-Sx.

## Discussion

The present study used a novel morphometry method to characterize fronto-striatal features associated with PD symptomatology. The results reveal differences in striatal shape and volume, and greater prefrontal cortical thickness, in subjects endorsing Cluster C PD-Sx (primarily OCPD), while no consistent pattern was observed in subjects endorsing Cluster B PD-Sx.

To our knowledge, the findings associated with Cluster C PD-Sx are the first cluster-level morphological findings to be reported in otherwise healthy individuals, although the subject was recently addressed in the context of substance use disorders (Albein-Urios et al., 2013, 2014; Moreno-López et al., 2014). It should be noted that nearly 90% of our Cluster C PD-Sx sample endorsed OCPD symptomatology, which is thought to stem from abnormalities in fronto-striatal circuitry (Fineberg et al., 2010), and consistent with this view, we observed smaller ventral striatum volumes, greater SA in the caudate tail, and greater PFC/OFC CT in our Cluster C PD-Sx sample. The finding of greater SA in caudate tail (**Figure 1**) is remarkably in line with a recent study using the same methodology to investigate OCD patients and their unaffected siblings, which identified a similar (albeit larger) effect as a candidate endophenotype likely reflecting genetic vulnerability (Shaw et al., 2015).

The cortical findings associated with Cluster C PD-Sx extend previous evidence that self-reported compulsive behavior correlates with greater PFC and OFC GM volume in adolescents (Montigny et al., 2013), and that individuals with social anxiety disorder exhibit greater CT in dorsolateral PFC (Brühl et al., 2014). Greater CT could reflect more complex and/or abundant circuitry (i.e., greater number of neurons, intracortical axons, dendrites, synaptic elements, or glia), perhaps due to inefficient pruning during maturation (Lotfipour et al., 2009), suggesting that this feature, in combination with possible enlargement in posterior caudate, could contribute to fronto-striatal hyper-connectivity, as has been suggested for OCD (Beucke et al., 2013; Shaw et al., 2015).

It is somewhat surprising that our analyses found no morphological features that were significantly associated with Cluster B PD-Sx, despite effect sizes similar to our Cluster C findings. Cluster B PDs involve emotion dysregulation, impulsive behavior, and aberrant motivation, often manifesting as criminality, aggression, self-harm, and substance use/relapse (Charney et al., 2010; Jahng et al., 2011), and these behaviors and traits (even when sub-clinical) have been strongly linked to the integrity of PFC and medial temporal structures (Matsuo et al., 2009; Yang and Raine, 2009; Bjork et al., 2010; Sato et al., 2012; Wallace et al., 2012; Montigny et al., 2013; Schilling et al., 2013; Castellanos-Ryan et al., 2014). Our failure to detect group differences in amygdala and hippocampus volumes was therefore unexpected (see Supplementary Materials for data and further discussion). Moreover, OFC abnormalities are consistently noted in clinically diagnosed BPD and ASPDs (Yang and Raine, 2009; Sato et al., 2012), but here, we only found preliminary evidence that lateral OFC SA may be lower in subjects with Cluster B PD-Sx (see Supplementary Materials). Interestingly, the evidence also points to enlargement of the posterior caudate in the present Cluster B PD-Sx sample (see Supplementary Materials), which would be consistent with previous evidence in PD-Sx individuals (Brambilla et al., 2004; Ersche et al., 2012; Glenn and Yang, 2012). Striatal enlargement is one of the cardinal findings in the stimulant use disorder literature, and although it could indicate gliosis (Chang et al., 2007; Kish, 2011; Mackey and Paulus, 2013), it has also been suggested that it could reflect a genetically determined predisposing feature (Ersche et al., 2012). Our findings would support the latter, but the study was not powered to definitively detect an effect.

Several limitations of the study should be noted. First, although cluster-based grouping of symptomatology allowed for investigation of consistencies across an entire cluster, this made the groups heterogeneous, and important nuances may have been lost. On the other hand, a significant percentage of PD patients show symptoms from more than one PD (DeJong et al., 1993), so that investigating shared features can identify more relevant cluster-level targets. The present sample size did not allow for further division than the cluster level, but future studies with larger samples will be able to address individual PDs, and evaluate effects that were observed only at trend levels in the present study. Second, assignment to the PD-Sx group in this study relied on self-report, as the Axis II questionnaire was not followed up with a clinical interview. Based on the questionnaire alone (which is sensitive, but not specific), subjects would have received more “diagnoses” than actually warranted, so that participants could not be diagnosed *per se*. Subjects who would have met vs. not met clinical criteria in the follow-up interview therefore had to be considered as a single group. Finally, many PDs are preceded by a history of abuse or other traumatic circumstances that could influence their trajectory, but this information was not collected in the present study. Similarly, no data on functional status or behavioral performance were collected, so that direct conclusions about functional relevance of morphological findings cannot be drawn. Although this limits our ability to determine the clinical relevance of our findings, the results can help guide future investigations.

These limitations notwithstanding, our results point to subcortical and cortical morphological features associated with Cluster C PD symptomatology that can guide future research into the etiology of PDs (especially OCPD). The findings further suggest that morphological features associated with PD-Sx should be taken into account in clinical studies of often-comorbid Axis I psychiatric disorders (e.g., substance use, mood, or anxiety disorders). Together, the findings present potential neurobiological targets to pursue in addressing PD symptomatology.

## Conflict of Interest Statement

The authors declare that the research was conducted in the absence of any commercial or financial relationships that could be construed as a potential conflict of interest.

## Footnotes

1 Note that this review combines ASPD and Psychopathy, which have similarities but also important distinctions.
