# Supplementary material for: Personality disorder symptomatology is associated with anomalies in striatal and prefrontal morphology
Source: Front Hum Neurosci. 2015 Aug 31;9:472. doi: 10.3389/fnhum.2015.00472 (PMC4553386; doi:10.3389/fnhum.2015.00472)
Supplement: Supplementary file 1 [file Table_1.DOCX]

***Supplementary Material***

**Personality disorder symptomatology is associated with
abnormalities in striatal and prefrontal morphology**

**Doris E. Payer^1, 2, 3^, Min Tae M. Park^4, 9^, Stephen J. Kish^2, 3^, Nathan J. Kolla^3, 6^, Jason P. Lerch^7, 8^, Isabelle Boileau^2, 3, *, †^, M. Mallar Chakravarty^4,5,†^**

^1^Addictions Program, Centre for Addiction and Mental Health, Toronto, ON, Canada

^2^Research Imaging Centre, Centre for Addiction and Mental Health, Toronto, ON, Canada

^3^Department of Psychiatry, University of Toronto, Toronto, ON, Canada

^4^Cerebral Imaging Centre, Douglas Mental Health University Institute, Verdun, QC, Canada

^5^Department of Psychiatry and Biomedical Engineering, McGill University; Montreal, QC Canada

^6^Complex Mental Illness Program, Forensic Service; CAMH, Toronto, ON, Canada

^7^Department of Medical Biophysics; University of Toronto, Toronto, ON, Canada

^8^Mouse Imaging Centre, Hospital for Sick Children, Toronto, ON, Canada

^9^Schulich School of Medicine and Dentistry, Western University, London, ON, Canada

^†^Co-Senior Author

*** Correspondence:** Isabelle Boileau, PhD; Centre for Addiction and Mental Health, Research Imaging Centre, 250 College St, Toronto, ON, M5T 1R8, Canada. [Isabelle.Boileau@camh.ca](mailto:Isabelle.Boileau@camh.ca)

1. **Supplementary Data**

The Supplementary Table below summarizes all subcortical and cortical effects detected in the atlas-derived ROIs.

- 1. **Regions associated with Cluster B symptomatology**

The omnibus MANCOVA analyzing subcortical ROI volumes detected effects in the Cluster B PD-Sx group in the hippocampus and posterior caudate at trend levels. Follow-up analyses directly testing for differences from control subjects revealed that these were driven by greater volumes in Cluster B PD-Sx subjects in the posterior caudate (right p=.022, left p=.058) and hippocampus (left p=.057). In cortical ROIs, the omnibus MANCOVA detected surface area effects for the Cluster B PD-Sx group in lateral orbitofrontal cortex, and follow-up comparison to Control subjects revealed that this reflected lower SA in subjects with Cluster B PD-Sx (right p=.036, left p=.068); in contrast, medial OFC showed greater SA (at trend levels; right p=.072).

The findings of greater hippocampal volume in the Cluster B PD-Sx group, and lack of any group difference in the amygdala, were surprising, as studies of BPD have reported smaller amygdala and hippocampus volumes so consistently that they have been proposed as a BPD endophenotype biomarker (Nunes et al., 2009; Ruocco et al., 2012). However, it is possible that the heterogeneity of endorsed Cluster B symptoms in our sample obscured this pattern, as the sample reporting BPD symptoms was relatively small (N=4) and non-BPD symptomatology could have masked the expected effect. ASPD/psychopathy and self-reported impulsivity have been associated with enlargement of subcortical structures, including the amygdala and striatum (Boccardi et al., 2011; Glenn and Yang, 2012; Ersche et al., 2013). In line with this, visual inspection of subcortical volumes in our Cluster B PD-Sx group, when divided by individual PDs, showed that subjects reporting BPD symptoms had smaller amygdala/hippocampus volumes than Control, while those reporting ASPD symptoms had greater volumes (data not shown; no statistical tests performed due to small sample sizes). It therefore appears that while amygdala/hippocampal shrinkage may be a reliable endophenotype for BPD, it may not be a shared feature across Cluster B PDs.

- 1. **Regions associated with Cluster C symptomatology**

In addition to the findings described in the main manuscript, comparing Cluster C PD-Sx to Control also revealed greater posterior caudate volumes (left, right p=.05), along with marginally greater hippocampal volume (left p=.057). In cortical ROIs, the omnibus MANCOVA identified primarily CT effects, particularly in PFC, OFC, and cingulate. In addition to the PFC/OFC effects described in the main manuscript, direct comparison of Cluster C PD-Sx to Control subjects revealed greater CT in the Cluster C PD-Sx group in the cingulate cortex (left p=.014, right p=.05). The comparison also revealed greater SA in medial OFC (right p=.008), but lower SA in lateral OFC (right p=.057).

PFC/OFC cortical thickening was reflected in the vertex-wise analysis at a relatively lenient threshold (Supplementary Figure), but did not survive FDR correction at q=.05.

- 1. **Common findings between Cluster B and Cluster C analyses**

It is interesting to note, as the far right column of the Supplementary Table illustrates, that several morphological features appear to overlap between subjects endorsing Cluster B and C PD-Sx (i.e., were found separately in both analyses compared to Control). These included greater posterior caudate volumes, consistent with a finding of striatal enlargement in individuals reporting both heightened impulsive and compulsive behaviour (Ersche et al., 2013), and greater SA in medial OFC but lower SA/greater CT in lateral OFC. Although not well-powered, we examined whether this overlap was driven by the 11 BC-comorbid subjects by comparing features between Cluster B-only (N=9), Cluster C-only (N=17), BC-comorbid (N=11), and Control subjects (N=35). The enlarged posterior caudate (relative to Control) was observed in both Cluster B-only (right p=.033, left p=.063) and C-only subjects (left p=.037). Similarly, the enlarged right medial OFC SA was observed in both Cluster B-only (p=.019) and C-only subjects (p=.016). On the other hand, the right lateral OFC SA effect was only observed in the BC-comorbid group, showing lower SA than Cluster B (p=.058), Cluster C (p=.01), and Control subjects (p=.003). The greater lateral OFC surface area was driven by Cluster C and BC-comorbid subjects (p=.071 and .046, respectively), and the hippocampus effect did not statistically differ between any of the sub-groups. These findings could form a basis for further investigation of dysfunctional personality traits across PDs, or vulnerability for development of PDs.

1. **Supplementary Figures and Tables**

**Supplementary Table 1.** Regions showing morphological differences between groups.

|  | **Omnibus Analysis^a^** | | **Cluster B vs. Control Analysis** | | | | **Omnibus Analysis^a^** | | **Cluster C vs. Control Analysis** | | | | **Common between Cluster B and C analyses** |
| --- | --- | --- | --- | --- | --- | --- | --- | --- | --- | --- | --- | --- | --- |
|  | **Cluster B Effect** | | **Marginal Mean (SE)** | |  | | **Cluster C Effect** | | **Marginal Mean (SE)** | |  | |  |
|  | **p** | **Cohen's d** | **Control (N=35)** | **Cluster B (N=20)** | **p** | **Cohen's d** | **p** | **Cohen's d** | **Control (N=35)** | **Cluster C (N=28)** | **p** | **Cohen's d** |  |
| **Subcortical Volume** |  |  |  |  |  |  |  |  |  |  |  |  |  |
| Posterior Caudate (R) | 0.10 | 0.42 | 1156.96 (26.05) | 1264.31 (35.27) | **0.022** | 0.56 | - | - | 1168.08 (30.86) | 1266.21 (35.01) | 0.051 | 0.53 | x |
| Posterior Caudate (L) | - | - | 1193.19 (24.91) | 1277.70 (33.72) | 0.058 | 0.56 | - | - | 1217.15 (27.81) | 1306.47 (31.55) | 0.053 | 0.53 | x |
| Ventral Striatum (R) | - | - | - | - | - | - | **0.01^b^** | 0.02 | 892.60 (11.35) | 841.60 (12.88) | **0.007^b^** | 0.75 |  |
| Ventral Striatum (L) | - | - | - | - | - | - | 0.06 | 0.12 | 1045.11 (13.74) | 999.48 (15.59) | **0.043** | 0.55 |  |
| Hippocampus (L) | 0.11 | 0.41 | 2092.85 (32.38) | 2203.28 (43.84) | 0.057 | 0.56 | - | - | 2109.21 (33.06) | 2212.26 (37.51) | 0.057 | 0.52 | x |
| **Cortical Thickness** |  |  |  |  |  |  |  |  |  |  |  |  |  |
| Lateral OFC (R) | - | - | 3.49 (0.03) | 3.59  (0.05) | 0.093 | 0.49 | **0.04** | 0.51 | 3.49 (0.04) | 3.62  (0.04) | **0.028^b^** | 0.60 | x |
| Medial OFC (L) | - | - | - | - | - | - | - | - | 3.32 (0.04) | 3.42  (0.04) | 0.087 | 0.46 |  |
| Inferior Frontal Gyrus (R) | - | - | - | - | - | - | **0.05** | 0.50 | 3.50 (0.03) | 3.56 (0.03) | 0.118 | 0.42 |  |
| Middle Frontal Gyrus (R) | - | - | - | - | - | - | **0.01^b^** | 0.72 | 9.52 (0.07) | 9.81  (0.08) | **0.016^b^** | 0.66 |  |
| Superior Frontal Gyrus (R) | - | - | - | - | - | - | **0.00^b^** | 0.80 | 3.41 (0.03) | 3.51  (0.03) | **0.017^b^** | 0.65 |  |
| Superior Frontal Gyrus (L) | - | - | - | - | - | - | **0.03** | 0.56 | 3.45 (0.03) | 3.53  (0.03) | 0.054 | 0.52 |  |
| Cingulate (R) | - | - | - | - | - | - | - | - | 3.36 (0.03) | 3.46  (0.04) | 0.051 | 0.53 |  |
| Cingulate (L) | - | - | - | - | - | - | 0.06 | 0.48 | 3.36 (0.03) | 3.48  (0.03) | **0.014** | 0.67 |  |
| **Surface Area** |  |  |  |  |  |  |  |  |  |  |  |  |  |
| Lateral OFC (R) | **0.02** | 0.60 | 1258.78 (37.49) | 1117.70 (50.74) | **0.036** | 0.62 | 0.09 | 0.44 | 1286.53 (36.22) | 1173.36 (41.09) | 0.057 | 0.52 | x |
| Lateral OFC (L) | 0.12 | 0.40 | 1277.90 (33.69) | 1167.85 (45.61) | 0.068 | 0.54 | - | - | - | - | - | - |  |
| Medial OFC (R) | - | - | 1931.17 (36.92) | 2049.89 (49.98) | 0.072 | 0.53 | - | - | 1931.28 (33.71) | 2081.29 (38.24) | **0.008** | 0.74 | x |


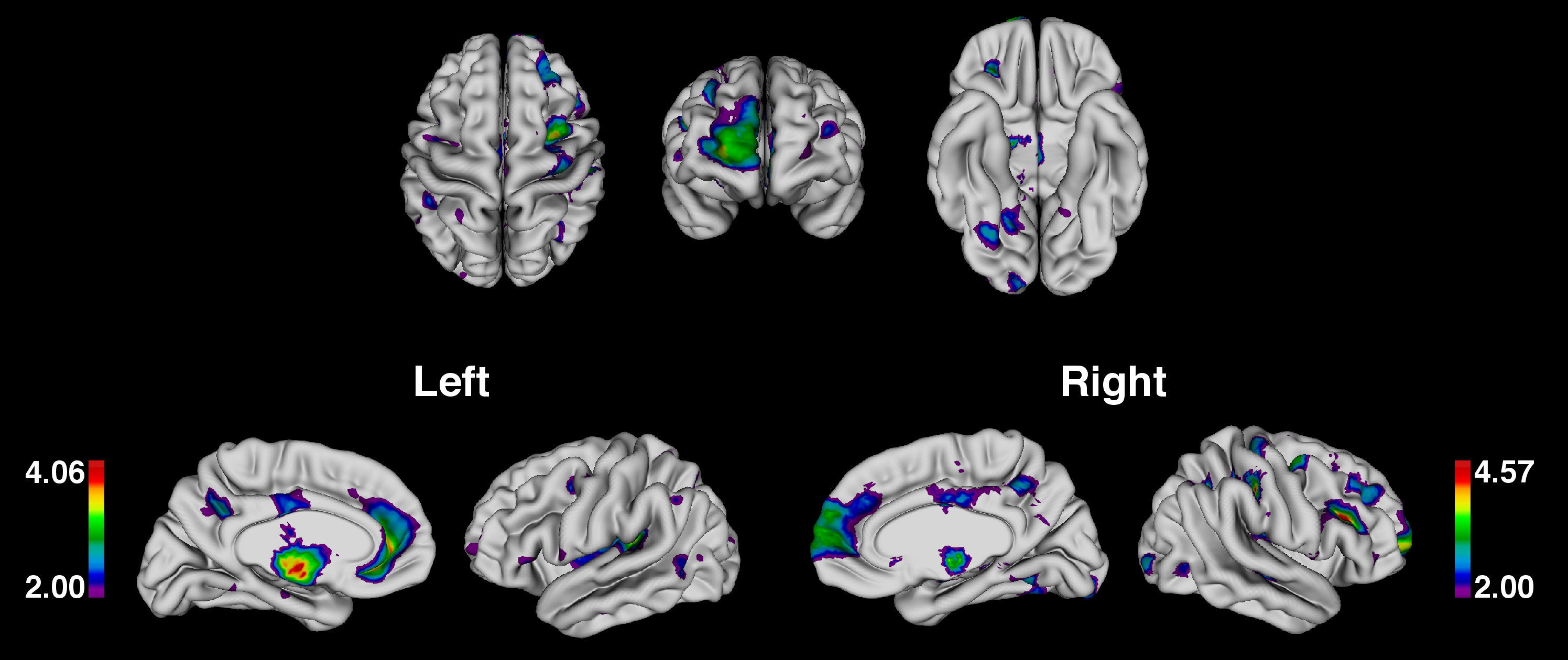
**Supplementary Figure 1**

**Supplementary Figure 1.** Significant associations between Cluster C symptomatology and greater cortical thickness, predominantly localized to right prefrontal cortex, as identified by vertex-wise analysis. No region survived FDR correction (q= 0.05), but patterns consistent with the region-of-interest-based analysis emerge at a more lenient threshold of t=2.0.
